# Supplementary material for: The impact of Covid-19 pandemic on overall well-being of practicing lawyers
Source: PLoS One. 2023 Mar 9;18(3):e0282836. doi: 10.1371/journal.pone.0282836 (PMC9997954; doi:10.1371/journal.pone.0282836)
Supplement: S1 File — (DOCX) [file pone.0282836.s001.docx]

# KBA Data Written up in paragraphs (not in tables or figures)

## Increased Stress and Difficulty

### Practice type and difficulty with practice due to pandemic

| **Crosstab** | | | | | | |
| --- | --- | --- | --- | --- | --- | --- |
|  | | | Practicing law more difficult_recoded | | | Total |
|  |  |  | Neutral | **Agree** | Disagree |  |
| Practice Type: Solo_Small_Large_Prosec_DPA | Solo Practice | Count | 43 | **363** | 67 | 473 |
|  |  | % within Practice Type: Solo_Small_Large_Prosec_DPA | 9.1% | **76.7%** | 14.2% | 100.0% |
|  | Small to Mid-Size Firm | Count | 27 | **466** | 92 | 585 |
|  |  | % within Practice Type: Solo_Small_Large_Prosec_DPA | 4.6% | **79.7%** | 15.7% | 100.0% |
|  | Large Firm | Count | 17 | **148** | 34 | 199 |
|  |  | % within Practice Type: Solo_Small_Large_Prosec_DPA | 8.5% | **74.4%** | 17.1% | 100.0% |
|  | Public Defender-DPA | Count | 4 | **41** | 2 | 47 |
|  |  | % within Practice Type: Solo_Small_Large_Prosec_DPA | 8.5% | **87.2%** | 4.3% | 100.0% |
|  | Prosecution | Count | 5 | **69** | 11 | 85 |
|  |  | % within Practice Type: Solo_Small_Large_Prosec_DPA | 5.9% | **81.2%** | 12.9% | 100.0% |
|  | All other types | Count | 47 | **211** | 40 | 298 |
|  |  | % within Practice Type: Solo_Small_Large_Prosec_DPA | 15.8% | **70.8%** | 13.4% | 100.0% |
| Total | | Count | 143 | **1298** | 246 | 1687 |
|  |  | % within Practice Type: Solo_Small_Large_Prosec_DPA | 8.5% | **76.9%** | 14.6% | 100.0% |

| **Chi-Square Tests** | | | |
| --- | --- | --- | --- |
|  | Value | df | Asymptotic Significance (2-sided) |
| Pearson Chi-Square | 38.229^a^ | 10 | <.001 |
| Likelihood Ratio | 37.847 | 10 | <.001 |
| Linear-by-Linear Association | 11.415 | 1 | <.001 |
| N of Valid Cases | 1687 |  |  |
| a. 1 cells (5.6%) have expected count less than 5. The minimum expected count is 3.98. | | | |

### Practice Type and increased stress due to pandemic

By practice type (i.e., solo, small-mid size firm, large firm, prosecutor, public defender/DPA, all other) more public defender/DPAs reported increased stress with their practice during the pandemic, (85.1%, *X^2^*(10, *N*=1689) =19.35, *p*=.036).

| Practice Type: Solo_Small_Large_Prosec_DPA * Practicing law more stressful_recoded Crosstabulation | | | | | | |
| --- | --- | --- | --- | --- | --- | --- |
|  | | | Practicing law more stressful_recoded | | | Total |
|  |  |  | Neutral | Agree | Disagree |  |
| Practice Type: Solo_Small_Large_Prosec_DPA | Solo Practice | Count | 56 | 348 | 70 | 474 |
|  |  | % within Practice Type: Solo_Small_Large_Prosec_DPA | 11.8% | 73.4% | 14.8% | 100.0% |
|  |  | % of Total | 3.3% | 20.6% | 4.1% | 28.1% |
|  | Small to Mid-Size Firm | Count | 50 | 450 | 85 | 585 |
|  |  | % within Practice Type: Solo_Small_Large_Prosec_DPA | 8.5% | 76.9% | 14.5% | 100.0% |
|  |  | % of Total | 3.0% | 26.6% | 5.0% | 34.6% |
|  | Large Firm | Count | 21 | 143 | 35 | 199 |
|  |  | % within Practice Type: Solo_Small_Large_Prosec_DPA | 10.6% | 71.9% | 17.6% | 100.0% |
|  |  | % of Total | 1.2% | 8.5% | 2.1% | 11.8% |
|  | Public Defender-DPA | Count | 5 | 40 | 2 | 47 |
|  |  | % within Practice Type: Solo_Small_Large_Prosec_DPA | 10.6% | 85.1% | 4.3% | 100.0% |
|  |  | % of Total | 0.3% | 2.4% | 0.1% | 2.8% |
|  | Prosecution | Count | 5 | 63 | 17 | 85 |
|  |  | % within Practice Type: Solo_Small_Large_Prosec_DPA | 5.9% | 74.1% | 20.0% | 100.0% |
|  |  | % of Total | 0.3% | 3.7% | 1.0% | 5.0% |
|  | All other types | Count | 46 | 213 | 40 | 299 |
|  |  | % within Practice Type: Solo_Small_Large_Prosec_DPA | 15.4% | 71.2% | 13.4% | 100.0% |
|  |  | % of Total | 2.7% | 12.6% | 2.4% | 17.7% |
| Total | | Count | 183 | 1257 | 249 | 1689 |
|  |  | % within Practice Type: Solo_Small_Large_Prosec_DPA | 10.8% | 74.4% | 14.7% | 100.0% |
|  |  | % of Total | 10.8% | 74.4% | 14.7% | 100.0% |

| Chi-Square Tests | | | |
| --- | --- | --- | --- |
|  | Value | df | Asymptotic Significance (2-sided) |
| Pearson Chi-Square | 19.348^a^ | 10 | .036 |
| Likelihood Ratio | 20.420 | 10 | .026 |
| Linear-by-Linear Association | 3.897 | 1 | .048 |
| N of Valid Cases | 1689 |  |  |
| a. 0 cells (0.0%) have expected count less than 5. The minimum expected count is 5.09. | | | |

## Resource Access

### Resources to help with stress by rural or urban

| Rural_Urban_recoded * Do you believe there are resources available to you to help deal with the stress of practice? Crosstabulation | | | | | |
| --- | --- | --- | --- | --- | --- |
|  | | | Do you believe there are resources available to you to help deal with the stress of practice? | | Total |
|  |  |  | Yes | No. |  |
| Rural_Urban_recoded | Urban_SmallCity | Count | 1033 | 220 | 1253 |
|  |  | % within Rural_Urban_recoded | 82.4% | 17.6% | 100.0% |
|  |  | % of Total | 69.6% | 14.8% | 84.4% |
|  | Rural | Count | 174 | 58 | 232 |
|  |  | % within Rural_Urban_recoded | 75.0% | 25.0% | 100.0% |
|  |  | % of Total | 11.7% | 3.9% | 15.6% |
| Total | | Count | 1207 | 278 | 1485 |
|  |  | % within Rural_Urban_recoded | 81.3% | 18.7% | 100.0% |
|  |  | % of Total | 81.3% | 18.7% | 100.0% |

| Chi-Square Tests | | | | | |
| --- | --- | --- | --- | --- | --- |
|  | Value | df | Asymptotic Significance (2-sided) | Exact Sig. (2-sided) | Exact Sig. (1-sided) |
| Pearson Chi-Square | 7.125^a^ | 1 | .008 |  |  |
| Continuity Correction^b^ | 6.645 | 1 | .010 |  |  |
| Likelihood Ratio | 6.704 | 1 | .010 |  |  |
| Fisher's Exact Test |  |  |  | .010 | .006 |
| Linear-by-Linear Association | 7.121 | 1 | .008 |  |  |
| N of Valid Cases | 1485 |  |  |  |  |
| a. 0 cells (0.0%) have expected count less than 5. The minimum expected count is 43.43. | | | | | |
| b. Computed only for a 2x2 table | | | | | |

### Resources by specific groups (criminal civil, other)

| Type of Law: Criminal_Civil_Other * Do you believe there are resources available to you to help deal with the stress of practice? Crosstabulation | | | | | |
| --- | --- | --- | --- | --- | --- |
|  | | | Do you believe there are resources available to you to help deal with the stress of practice? | | Total |
|  |  |  | Yes | No. |  |
| Type of Law: Criminal_Civil_Other | Criminal Law | Count | 131 | 49 | 180 |
|  |  | % within Type of Law: Criminal_Civil_Other | 72.8% | 27.2% | 100.0% |
|  |  | % of Total | 8.9% | 3.3% | 12.2% |
|  | Civil Litigation | Count | 394 | 94 | 488 |
|  |  | % within Type of Law: Criminal_Civil_Other | 80.7% | 19.3% | 100.0% |
|  |  | % of Total | 26.6% | 6.4% | 33.0% |
|  | All other types of law | Count | 680 | 131 | 811 |
|  |  | % within Type of Law: Criminal_Civil_Other | 83.8% | 16.2% | 100.0% |
|  |  | % of Total | 46.0% | 8.9% | 54.8% |
| Total | | Count | 1205 | 274 | 1479 |
|  |  | % within Type of Law: Criminal_Civil_Other | 81.5% | 18.5% | 100.0% |
|  |  | % of Total | 81.5% | 18.5% | 100.0% |

| Chi-Square Tests | | | |
| --- | --- | --- | --- |
|  | Value | df | Asymptotic Significance (2-sided) |
| Pearson Chi-Square | 12.220^a^ | 2 | .002 |
| Likelihood Ratio | 11.450 | 2 | .003 |
| Linear-by-Linear Association | 11.172 | 1 | <.001 |
| N of Valid Cases | 1479 |  |  |
| a. 0 cells (0.0%) have expected count less than 5. The minimum expected count is 33.35. | | | |

### Resources by Practice Type

| **Crosstab** | | | | | |
| --- | --- | --- | --- | --- | --- |
|  | | | Do you believe there are resources available to you to help deal with the stress of practice? | | Total |
|  |  |  | Yes | **No.** |  |
| Practice Type: Solo_Small_Large_Prosec_DPA | Solo Practice | Count | 319 | **103** | 422 |
|  |  | % within Practice Type: Solo_Small_Large_Prosec_DPA | 75.6% | **24.4%** | 100.0% |
|  | Small to Mid-Size Firm | Count | 435 | **91** | 526 |
|  |  | % within Practice Type: Solo_Small_Large_Prosec_DPA | 82.7% | **17.3%** | 100.0% |
|  | Large Firm | Count | 155 | **22** | 177 |
|  |  | % within Practice Type: Solo_Small_Large_Prosec_DPA | 87.6% | **12.4%** | 100.0% |
|  | Public Defender-DPA | Count | 28 | **12** | 40 |
|  |  | % within Practice Type: Solo_Small_Large_Prosec_DPA | 70.0% | **30.0%** | 100.0% |
|  | Prosecution | Count | 50 | **19** | 69 |
|  |  | % within Practice Type: Solo_Small_Large_Prosec_DPA | 72.5% | **27.5%** | 100.0% |
|  | All other types | Count | 229 | **37** | 266 |
|  |  | % within Practice Type: Solo_Small_Large_Prosec_DPA | 86.1% | **13.9%** | 100.0% |
| Total | | Count | 1216 | **284** | 1500 |
|  |  | % within Practice Type: Solo_Small_Large_Prosec_DPA | 81.1% | **18.9%** | 100.0% |

| **Chi-Square Tests** | | | |
| --- | --- | --- | --- |
|  | Value | df | Asymptotic Significance (2-sided) |
| **Pearson Chi-Square** | **24.924^a^** | **5** | **<.001** |
| Likelihood Ratio | 24.615 | 5 | <.001 |
| Linear-by-Linear Association | 5.247 | 1 | .022 |
| N of Valid Cases | 1500 |  |  |
| a. 0 cells (0.0%) have expected count less than 5. The minimum expected count is 7.57. | | | |

### Have Someone to Talk To About Stress by Practice Type

| **Crosstab** | | | | | |
| --- | --- | --- | --- | --- | --- |
|  | | | Do you have someone you can talk to about the stress of practice? | | Total |
|  |  |  | Yes | **No opinion or prefer not to answer** |  |
| Practice Type: Solo_Small_Large_Prosec_DPA | Solo Practice | Count | 355 | **54** | 409 |
|  |  | % within Practice Type: Solo_Small_Large_Prosec_DPA | 86.8% | **13.2%** | 100.0% |
|  | Small to Mid-Size Firm | Count | 486 | **51** | 537 |
|  |  | % within Practice Type: Solo_Small_Large_Prosec_DPA | 90.5% | **9.5%** | 100.0% |
|  | Large Firm | Count | 167 | **14** | 181 |
|  |  | % within Practice Type: Solo_Small_Large_Prosec_DPA | 92.3% | **7.7%** | 100.0% |
|  | Public Defender-DPA | Count | 34 | **10** | 44 |
|  |  | % within Practice Type: Solo_Small_Large_Prosec_DPA | 77.3% | **22.7%** | 100.0% |
|  | Prosecution | Count | 60 | **11** | 71 |
|  |  | % within Practice Type: Solo_Small_Large_Prosec_DPA | 84.5% | **15.5%** | 100.0% |
|  | All other types | Count | 239 | **38** | 277 |
|  |  | % within Practice Type: Solo_Small_Large_Prosec_DPA | 86.3% | **13.7%** | 100.0% |
| Total | | Count | 1341 | **178** | 1519 |
|  |  | % within Practice Type: Solo_Small_Large_Prosec_DPA | 88.3% | **11.7%** | 100.0% |

| **Chi-Square Tests** | | | |
| --- | --- | --- | --- |
|  | Value | df | Asymptotic Significance (2-sided) |
| Pearson Chi-Square | 13.412^a^ | 5 | .020 |
| Likelihood Ratio | 12.780 | 5 | .026 |
| Linear-by-Linear Association | 1.617 | 1 | .204 |
| N of Valid Cases | 1519 |  |  |
| a. 0 cells (0.0%) have expected count less than 5. The minimum expected count is 5.16. | | | |

## Work Satisfaction

### Average and Median Ratings Judges-and Practice Type

|  | | | |
| --- | --- | --- | --- |
| On a scale of 0-100, with 0 being completely unsatisfied and 100 being completely satisfied, what was/is your satisfaction with the practice of law: - | | Prior to the Pandemic | Currently |
| Solo practice | Mean | 73.10 | 58.37 |
|  | N | 1046 | 997 |
|  | Std. Deviation | 20.381 | 25.514 |
|  | **Grouped Median** | **79.42** | **60.59** |
|  | Range | 100 | 100 |
| Small to Mid-size Firm | Mean | 71.63 | 60.97 |
|  | N | 234 | 230 |
|  | Std. Deviation | 18.891 | 22.309 |
|  | **Grouped Median** | **75.54** | **64.71** |
|  | Range | 100 | 100 |
| Large Firm | Mean | 72.97 | 65.73 |
|  | N | 229 | 227 |
|  | Std. Deviation | 19.143 | 22.058 |
|  | **Grouped Median** | **79.15** | **70.09** |
|  | Range | 100 | 97 |
| Counsel, DPA, Prosecuter, Clerks, Other | Mean | 71.26 | 60.61 |
|  | N | 489 | 474 |
|  | Std. Deviation | 20.723 | 24.731 |
|  | **Grouped Median** | **75.24** | **65.45** |
|  | Range | 100 | 100 |
| Judges | Mean | 76.19 | 59.42 |
|  | N | 47 | 45 |
|  | Std. Deviation | 23.635 | 28.728 |
|  | **Grouped Median** | **81.00** | **62.00** |
|  | Range | 100 | 100 |
| Total | Mean | 72.55 | 60.08 |
|  | N | 2045 | 1973 |
|  | Std. Deviation | 20.248 | 24.754 |
|  | Grouped Median | 79.09 | 62.95 |
|  | Range | 100 | 100 |

| ANOVA Table | | | | | | | |
| --- | --- | --- | --- | --- | --- | --- | --- |
|  | | | Sum of Squares | df | Mean Square | F | Sig. |
| Prior to the Pandemic * | Between Groups | (Combined) | 1996.903 | 4 | 499.226 | 1.218 | .301 |
|  | Within Groups | | 836039.309 | 2040 | 409.823 |  |  |
|  | Total | | 838036.212 | 2044 |  |  |  |
| Currently | Between Groups | (Combined) | 10473.803 | 4 | 2618.451 | 4.302 | .002 |
|  | Within Groups | | 1197892.565 | 1968 | 608.685 |  |  |
|  | Total | | 1208366.368 | 1972 |  |  |  |

|  | | | | | |  |  |  |  |  |  |  |
| --- | --- | --- | --- | --- | --- | --- | --- | --- | --- | --- | --- | --- |
| \| Practice Type \| Median Work Satisfaction Ratings (Scale 0-100) \| \| \| \| --- \| --- \| --- \| --- \| \|  \| Before Pandemic \| At Present \| Difference \| \| Solo Practice \| 79.42 \| 60.59 \| -18.83 \| \| Small to Mid-size Firm \| 75.54 \| 64.71 \| -10.83 \| \| Large Firm \| 79.15 \| 70.09 \| -9.06 \| \| Public Defender, DPA, Prosecution, Other \| 75.24 \| 65.45 \| -9.79 \| \| Judicial \| 81.00 \| 62.00 \| -19.00 \| | | | | | |  |  |  |  |  |  |  |
|  | |  | |  | |  |  |  |  |  |  |  |
| Years of practice and job satisfaction ratings  \| Descriptives On a scale of 0-100, with 0 being completely unsatisfied and 100 being completely satisfied, what was/is your satisfaction with the practice of law: - \| \| \| \| \| \| \| \| \| \| \| --- \| --- \| --- \| --- \| --- \| --- \| --- \| --- \| --- \| --- \| \|  \| \| N \| Mean \| Std. Deviation \| Std. Error \| 95% Confidence Interval for Mean \| \| Minimum \| Maximum \| \| Lower Bound \| Upper Bound \| \| Prior to the Pandemic \| less than 2 years \| 76 \| 73.05 \| 19.431 \| 2.229 \| 68.61 \| 77.49 \| 0 \| 100 \| \| 3-5 years \| 131 \| 71.32 \| 19.396 \| 1.695 \| 67.97 \| 74.67 \| 10 \| 100 \| \| 6-10 years \| 227 \| 70.88 \| 18.324 \| 1.216 \| 68.48 \| 73.28 \| 0 \| 100 \| \| 11-15 years \| 274 \| 67.64 \| 20.353 \| 1.230 \| 65.21 \| 70.06 \| 0 \| 100 \| \| 16-20 years \| 228 \| 71.47 \| 20.639 \| 1.367 \| 68.78 \| 74.17 \| 7 \| 100 \| \| 21-25 years \| 261 \| 69.36 \| 20.359 \| 1.260 \| 66.87 \| 71.84 \| 5 \| 100 \| \| over 25 years \| 839 \| 76.04 \| 20.184 \| .697 \| 74.67 \| 77.40 \| 0 \| 100 \| \| Total \| 2036 \| 72.55 \| 20.228 \| .448 \| 71.67 \| 73.43 \| 0 \| 100 \| \| Currently \| less than 2 years \| 73 \| 50.63 \| 27.710 \| 3.243 \| 44.16 \| 57.10 \| 0 \| 100 \| \| 3-5 years \| 131 \| 59.45 \| 24.408 \| 2.133 \| 55.23 \| 63.67 \| 0 \| 100 \| \| 6-10 years \| 222 \| 58.56 \| 24.344 \| 1.634 \| 55.34 \| 61.78 \| 0 \| 100 \| \| 11-15 years \| 269 \| 58.34 \| 23.353 \| 1.424 \| 55.53 \| 61.14 \| 0 \| 100 \| \| 16-20 years \| 224 \| 59.04 \| 24.656 \| 1.647 \| 55.79 \| 62.28 \| 0 \| 100 \| \| 21-25 years \| 255 \| 59.20 \| 22.768 \| 1.426 \| 56.39 \| 62.01 \| 0 \| 100 \| \| over 25 years \| 791 \| 62.68 \| 25.458 \| .905 \| 60.90 \| 64.45 \| 0 \| 100 \| \| Total \| 1965 \| 60.09 \| 24.747 \| .558 \| 58.99 \| 61.18 \| 0 \| 100 \|  \| ANOVA \| \| \| \| \| \| \| \| --- \| --- \| --- \| --- \| --- \| --- \| --- \| \|  \| \| Sum of Squares \| df \| Mean Square \| F \| Sig. \| \| Prior to the Pandemic \| Between Groups \| 20588.939 \| 6 \| 3431.490 \| 8.574 \| <.001 \| \| Within Groups \| 812047.247 \| 2029 \| 400.220 \|  \|  \| \| Total \| 832636.186 \| 2035 \|  \|  \|  \| \| Currently \| Between Groups \| 13676.654 \| 6 \| 2279.442 \| 3.753 \| .001 \| \| Within Groups \| 1189107.582 \| 1958 \| 607.307 \|  \|  \| \| Total \| 1202784.236 \| 1964 \|  \|  \|  \|   Satisfaction Ratings - On a scale of 0-100, with 0 being completely unsatisfied and 100 being completely satisfied, what was/is your satisfaction with the practice of law: -   \| Years of practice \| Pre-pandemic \| Present \|  \| Difference \| \| --- \| --- \| --- \| --- \| --- \| \| less than 2 years \| 73.05 \| 50.63 \|  \| 22.42 \| \| 3-5 years \| 71.32 \| 59.45 \|  \| 11.87 \| \| 6-10 years \| 70.88 \| 58.56 \|  \| 12.32 \| \| 11-15 years \| 67.64 \| 58.34 \|  \| 9.3 \| \| 16-20 years \| 71.47 \| 59.04 \|  \| 12.43 \| \| 21-25 years \| 69.36 \| 59.20 \|  \| 10.16 \| \| over 25 years \| 76.04 \| 62.68 \|  \| 13.36 \| \| Total \| 72.55 \| 60.09 \|  \|  \|  Impact of Pandemic Pos-neutral-neg by years of practice How long have you been licensed to practice law? * Overall, the impact of the pandemic on my practice was: Crosstabulation | | | | | | | | | | | |  |
|  | | | | | Overall, the impact of the pandemic on my practice was: | | | | | Total | |  |
|  |  |  |  |  | Very negative | Somewhat negative | Neutral | Somewhat positive | Very Positive | |  | |
| How long have you been licensed to practice law? | less than 2 years | | Count | | 21 | 29 | 15 | 5 | 4 | | 74 | |
|  |  |  | % within How long have you been licensed to practice law? | | 28.4% | 39.2% | 20.3% | 6.8% | 5.4% | | 100.0% | |
|  | 3-5 years | | Count | | 16 | 59 | 36 | 13 | 4 | | 128 | |
|  |  |  | % within How long have you been licensed to practice law? | | 12.5% | 46.1% | 28.1% | 10.2% | 3.1% | | 100.0% | |
|  | 6-10 years | | Count | | 36 | 106 | 47 | 27 | 11 | | 227 | |
|  |  |  | % within How long have you been licensed to practice law? | | 15.9% | 46.7% | 20.7% | 11.9% | 4.8% | | 100.0% | |
|  | 11-15 years | | Count | | 44 | 132 | 53 | 30 | 10 | | 269 | |
|  |  |  | % within How long have you been licensed to practice law? | | 16.4% | 49.1% | 19.7% | 11.2% | 3.7% | | 100.0% | |
|  | 16-20 years | | Count | | 41 | 101 | 50 | 25 | 9 | | 226 | |
|  |  |  | % within How long have you been licensed to practice law? | | 18.1% | 44.7% | 22.1% | 11.1% | 4.0% | | 100.0% | |
|  | 21-25 years | | Count | | 47 | 120 | 59 | 26 | 6 | | 258 | |
|  |  |  | % within How long have you been licensed to practice law? | | 18.2% | 46.5% | 22.9% | 10.1% | 2.3% | | 100.0% | |
|  | over 25 years | | Count | | 175 | 379 | 164 | 77 | 18 | | 813 | |
|  |  |  | % within How long have you been licensed to practice law? | | 21.5% | 46.6% | 20.2% | 9.5% | 2.2% | | 100.0% | |
| Total | | | Count | | 380 | 926 | 424 | 203 | 62 | | 1995 | |
|  |  |  | % within How long have you been licensed to practice law? | | 19.0% | 46.4% | 21.3% | 10.2% | 3.1% | | 100.0% | |

| Chi-Square Tests | | | |
| --- | --- | --- | --- |
|  | Value | df | Asymptotic Significance (2-sided) |
| Pearson Chi-Square | 26.041^a^ | 24 | .351 |
| Likelihood Ratio | 25.545 | 24 | .377 |
| Linear-by-Linear Association | 6.480 | 1 | .011 |
| N of Valid Cases | 1995 |  |  |
| a. 2 cells (5.7%) have expected count less than 5. The minimum expected count is 2.30. | | | |

| Symmetric Measures | | | | | |
| --- | --- | --- | --- | --- | --- |
|  | | Value | Asymptotic Standard Error^a^ | Approximate T^b^ | Approximate Significance |
| Interval by Interval | Pearson's R | -.057 | .023 | -2.549 | .011^c^ |
| Ordinal by Ordinal | Spearman Correlation | -.060 | .022 | -2.702 | .007^c^ |
| N of Valid Cases | | 1995 |  |  |  |
| a. Not assuming the null hypothesis. | | | | | |
| b. Using the asymptotic standard error assuming the null hypothesis. | | | | | |
| c. Based on normal approximation. | | | | | |

### Overall Impact -Years of Practice Combined

| Years licensed to practice_R * Overall impact on practice - positive-neutral-negative Crosstabulation | | | | | | | | | | | | | | | |
| --- | --- | --- | --- | --- | --- | --- | --- | --- | --- | --- | --- | --- | --- | --- | --- |
|  | | | | | | | | | Overall impact on practice - positive-neutral-negative | | | | | | Total |
|  |  |  |  |  |  |  |  |  | Negative | Neutral | | | Positive | |  |
| Years licensed to practice_R | | <=5 years | | Count | | | | | **125** | 51 | | | 26 | | 202 |
|  |  |  |  | % within Years licensed to practice_R | | | | | **61.9%** | 25.2% | | | 12.9% | | 100.0% |
|  |  | 6-15 years | | Count | | | | | **318** | 100 | | | 78 | | 496 |
|  |  |  |  | % within Years licensed to practice_R | | | | | **64.1%** | 20.2% | | | 15.7% | | 100.0% |
|  |  | 16-25 years | | Count | | | | | **309** | 109 | | | 66 | | 484 |
|  |  |  |  | % within Years licensed to practice_R | | | | | **63.8%** | 22.5% | | | 13.6% | | 100.0% |
|  |  | >25 years | | Count | | | | | **554** | 164 | | | 95 | | 813 |
|  |  |  |  | % within Years licensed to practice_R | | | | | **68.1%** | 20.2% | | | 11.7% | | 100.0% |
| Total | | | | Count | | | | | 1306 | 424 | | | 265 | | 1995 |
|  |  |  |  | % within Years licensed to practice_R | | | | | 65.5% | 21.3% | | | 13.3% | | 100.0% |
| Chi-Square Tests | | | | | | | | | | |  |  |  |  |  |
|  | | | Value | | df | | Asymptotic Significance (2-sided) | | | |  |  |  |  |  |
| Pearson Chi-Square | | | 8.089^a^ | | 6 | | .232 | | | |  |  |  |  |  |
| Likelihood Ratio | | | 7.948 | | 6 | | .242 | | | |  |  |  |  |  |
| Linear-by-Linear Association | | | 4.136 | | 1 | | .042 | | | |  |  |  |  |  |
| N of Valid Cases | | | 1995 | |  | |  | | | |  |  |  |  |  |
| a. 0 cells (0.0%) have expected count less than 5. The minimum expected count is 26.83. | | | | | | | | | | |  |  |  |  |  |
| Symmetric Measures | | | | | | | | | | | | | | | |
|  | | | | | | Value | | Asymptotic Standard Error^a^ | | | | Approximate T^b^ | | Approximate Significance | |
| Interval by Interval | Pearson's R | | | | | -.046 | | .022 | | | | -2.035 | | .042^c^ | |
| Ordinal by Ordinal | Spearman Correlation | | | | | -.047 | | .022 | | | | -2.116 | | .035^c^ | |
| N of Valid Cases | | | | | | 1995 | |  | | | |  | |  | |
| a. Not assuming the null hypothesis. | | | | | | | | | | | | | | | |
| b. Using the asymptotic standard error assuming the null hypothesis. | | | | | | | | | | | | | | | |
| c. Based on normal approximation. | | | | | | | | | | | | | | | |

## Relationships

### Positive Relationships by Practice Type

| Crosstab | | | | | | |
| --- | --- | --- | --- | --- | --- | --- |
|  | | | Hard to maintain positive relationships with other lawyers_recoded | | | Total |
|  |  |  | Neutral | Agree | Disagree |  |
| Practice Type: Solo_Small_Large_Prosec_DPA | Solo Practice | Count | 90 | 267 | 117 | 474 |
|  |  | % within Practice Type: Solo_Small_Large_Prosec_DPA | 19.0% | 56.3% | 24.7% | 100.0% |
|  | Small to Mid-Size Firm | Count | 79 | 348 | 158 | 585 |
|  |  | % within Practice Type: Solo_Small_Large_Prosec_DPA | 13.5% | 59.5% | 27.0% | 100.0% |
|  | Large Firm | Count | 26 | 131 | 42 | 199 |
|  |  | % within Practice Type: Solo_Small_Large_Prosec_DPA | 13.1% | 65.8% | 21.1% | 100.0% |
|  | Public Defender-DPA | Count | 5 | 31 | 11 | 47 |
|  |  | % within Practice Type: Solo_Small_Large_Prosec_DPA | 10.6% | 66.0% | 23.4% | 100.0% |
|  | Prosecution | Count | 11 | 41 | 33 | 85 |
|  |  | % within Practice Type: Solo_Small_Large_Prosec_DPA | 12.9% | 48.2% | 38.8% | 100.0% |
|  | All other types | Count | 60 | 166 | 72 | 298 |
|  |  | % within Practice Type: Solo_Small_Large_Prosec_DPA | 20.1% | 55.7% | 24.2% | 100.0% |
| Total | | Count | 271 | 984 | 433 | 1688 |
|  |  | % within Practice Type: Solo_Small_Large_Prosec_DPA | 16.1% | 58.3% | 25.7% | 100.0% |

| Chi-Square Tests | | | |
| --- | --- | --- | --- |
|  | Value | df | Asymptotic Significance (2-sided) |
| Pearson Chi-Square | 23.461^a^ | 10 | .009 |
| Likelihood Ratio | 22.773 | 10 | .012 |
| Linear-by-Linear Association | 1.490 | 1 | .222 |
| N of Valid Cases | 1688 |  |  |
| a. 0 cells (0.0%) have expected count less than 5. The minimum expected count is 7.55. | | | |

### Interferes with Home Life by Type of Practice

| Crosstab | | | | | | |
| --- | --- | --- | --- | --- | --- | --- |
|  | | | Demands of practice interfere with home life_recoded | | | Total |
|  |  |  | Neutral | Agree | Disagree |  |
| Practice Type: Solo_Small_Large_Prosec_DPA | Solo Practice | Count | 73 | 238 | 162 | 473 |
|  |  | % within Practice Type: Solo_Small_Large_Prosec_DPA | 15.4% | 50.3% | 34.2% | 100.0% |
|  | Small to Mid-Size Firm | Count | 71 | 353 | 161 | 585 |
|  |  | % within Practice Type: Solo_Small_Large_Prosec_DPA | 12.1% | 60.3% | 27.5% | 100.0% |
|  | Large Firm | Count | 15 | 141 | 43 | 199 |
|  |  | % within Practice Type: Solo_Small_Large_Prosec_DPA | 7.5% | 70.9% | 21.6% | 100.0% |
|  | Public Defender-DPA | Count | 6 | 31 | 10 | 47 |
|  |  | % within Practice Type: Solo_Small_Large_Prosec_DPA | 12.8% | 66.0% | 21.3% | 100.0% |
|  | Prosecution | Count | 13 | 43 | 29 | 85 |
|  |  | % within Practice Type: Solo_Small_Large_Prosec_DPA | 15.3% | 50.6% | 34.1% | 100.0% |
|  | All other types | Count | 56 | 159 | 84 | 299 |
|  |  | % within Practice Type: Solo_Small_Large_Prosec_DPA | 18.7% | 53.2% | 28.1% | 100.0% |
| Total | | Count | 234 | 965 | 489 | 1688 |
|  |  | % within Practice Type: Solo_Small_Large_Prosec_DPA | 13.9% | 57.2% | 29.0% | 100.0% |

| Chi-Square Tests | | | |
| --- | --- | --- | --- |
|  | Value | df | Asymptotic Significance (2-sided) |
| Pearson Chi-Square | 37.177^a^ | 10 | <.001 |
| Likelihood Ratio | 37.711 | 10 | <.001 |
| Linear-by-Linear Association | 3.270 | 1 | .071 |
| N of Valid Cases | 1688 |  |  |
| a. 0 cells (0.0%) have expected count less than 5. The minimum expected count is 6.52. | | | |

### Strain on Family by Practice Type

| Crosstab | | | | | | |
| --- | --- | --- | --- | --- | --- | --- |
|  | | | Practice of law has put strain on my family_recoded | | | Total |
|  |  |  | Neutral | Agree | Disagree |  |
| Practice Type: Solo_Small_Large_Prosec_DPA | Solo Practice | Count | 84 | 210 | 178 | 472 |
|  |  | % within Practice Type: Solo_Small_Large_Prosec_DPA | 17.8% | 44.5% | 37.7% | 100.0% |
|  | Small to Mid-Size Firm | Count | 81 | 285 | 218 | 584 |
|  |  | % within Practice Type: Solo_Small_Large_Prosec_DPA | 13.9% | 48.8% | 37.3% | 100.0% |
|  | Large Firm | Count | 37 | 101 | 61 | 199 |
|  |  | % within Practice Type: Solo_Small_Large_Prosec_DPA | 18.6% | 50.8% | 30.7% | 100.0% |
|  | Public Defender-DPA | Count | 8 | 26 | 12 | 46 |
|  |  | % within Practice Type: Solo_Small_Large_Prosec_DPA | 17.4% | 56.5% | 26.1% | 100.0% |
|  | Prosecution | Count | 9 | 37 | 39 | 85 |
|  |  | % within Practice Type: Solo_Small_Large_Prosec_DPA | 10.6% | 43.5% | 45.9% | 100.0% |
|  | All other types | Count | 74 | 123 | 102 | 299 |
|  |  | % within Practice Type: Solo_Small_Large_Prosec_DPA | 24.7% | 41.1% | 34.1% | 100.0% |
| Total | | Count | 293 | 782 | 610 | 1685 |
|  |  | % within Practice Type: Solo_Small_Large_Prosec_DPA | 17.4% | 46.4% | 36.2% | 100.0% |

| Chi-Square Tests | | | |
| --- | --- | --- | --- |
|  | Value | df | Asymptotic Significance (2-sided) |
| Pearson Chi-Square | 26.861^a^ | 10 | .003 |
| Likelihood Ratio | 26.489 | 10 | .003 |
| Linear-by-Linear Association | 6.034 | 1 | .014 |
| N of Valid Cases | 1685 |  |  |
| a. 0 cells (0.0%) have expected count less than 5. The minimum expected count is 8.00. | | | |

### Stress from work comes home with me by practice type

| Crosstab | | | | | | |
| --- | --- | --- | --- | --- | --- | --- |
|  | | | Stress from work comes home with me_recoded | | | Total |
|  |  |  | Neutral | Agree | Disagree |  |
| Practice Type: Solo_Small_Large_Prosec_DPA | Solo Practice | Count | 47 | 272 | 153 | 472 |
|  |  | % within Practice Type: Solo_Small_Large_Prosec_DPA | 10.0% | 57.6% | 32.4% | 100.0% |
|  | Small to Mid-Size Firm | Count | 37 | 383 | 165 | 585 |
|  |  | % within Practice Type: Solo_Small_Large_Prosec_DPA | 6.3% | 65.5% | 28.2% | 100.0% |
|  | Large Firm | Count | 15 | 142 | 42 | 199 |
|  |  | % within Practice Type: Solo_Small_Large_Prosec_DPA | 7.5% | 71.4% | 21.1% | 100.0% |
|  | Public Defender-DPA | Count | 2 | 37 | 8 | 47 |
|  |  | % within Practice Type: Solo_Small_Large_Prosec_DPA | 4.3% | 78.7% | 17.0% | 100.0% |
|  | Prosecution | Count | 9 | 54 | 21 | 84 |
|  |  | % within Practice Type: Solo_Small_Large_Prosec_DPA | 10.7% | 64.3% | 25.0% | 100.0% |
|  | All other types | Count | 36 | 195 | 67 | 298 |
|  |  | % within Practice Type: Solo_Small_Large_Prosec_DPA | 12.1% | 65.4% | 22.5% | 100.0% |
| Total | | Count | 146 | 1083 | 456 | 1685 |
|  |  | % within Practice Type: Solo_Small_Large_Prosec_DPA | 8.7% | 64.3% | 27.1% | 100.0% |

| Chi-Square Tests | | | |
| --- | --- | --- | --- |
|  | Value | df | Asymptotic Significance (2-sided) |
| Pearson Chi-Square | 28.972^a^ | 10 | .001 |
| Likelihood Ratio | 29.390 | 10 | .001 |
| Linear-by-Linear Association | 9.261 | 1 | .002 |
| N of Valid Cases | 1685 |  |  |
| a. 1 cells (5.6%) have expected count less than 5. The minimum expected count is 4.07. | | | |

## Mental Health Impact

### Frequent Depression by Practice Type

| Crosstab | | | | | | | | | | |
| --- | --- | --- | --- | --- | --- | --- | --- | --- | --- | --- |
|  | | | | | | | Depressed frequently_recoded | | | Total |
|  |  |  |  |  |  |  | Neutral | Agree | Disagree |  |
| Practice Type: Solo_Small_Large_Prosec_DPA | | Solo Practice | | Count | | | 77 | 131 | 266 | 474 |
|  |  |  |  | % within Practice Type: Solo_Small_Large_Prosec_DPA | | | 16.2% | 27.6% | 56.1% | 100.0% |
|  |  | Small to Mid-Size Firm | | Count | | | 79 | 163 | 342 | 584 |
|  |  |  |  | % within Practice Type: Solo_Small_Large_Prosec_DPA | | | 13.5% | 27.9% | 58.6% | 100.0% |
|  |  | Large Firm | | Count | | | 36 | 43 | 120 | 199 |
|  |  |  |  | % within Practice Type: Solo_Small_Large_Prosec_DPA | | | 18.1% | 21.6% | 60.3% | 100.0% |
|  |  | Public Defender-DPA | | Count | | | 6 | 23 | 18 | 47 |
|  |  |  |  | % within Practice Type: Solo_Small_Large_Prosec_DPA | | | 12.8% | 48.9% | 38.3% | 100.0% |
|  |  | Prosecution | | Count | | | 10 | 24 | 51 | 85 |
|  |  |  |  | % within Practice Type: Solo_Small_Large_Prosec_DPA | | | 11.8% | 28.2% | 60.0% | 100.0% |
|  |  | All other types | | Count | | | 48 | 94 | 154 | 296 |
|  |  |  |  | % within Practice Type: Solo_Small_Large_Prosec_DPA | | | 16.2% | 31.8% | 52.0% | 100.0% |
| Total | | | | Count | | | 256 | 478 | 951 | 1685 |
|  |  |  |  | % within Practice Type: Solo_Small_Large_Prosec_DPA | | | 15.2% | 28.4% | 56.4% | 100.0% |
| Chi-Square Tests | | | | | |  |  |  |  |  |
|  | Value | | df | | Asymptotic Significance (2-sided) |  |  |  |  |  |
| Pearson Chi-Square | 20.054^a^ | | 10 | | .029 |  |  |  |  |  |
| Likelihood Ratio | 19.429 | | 10 | | .035 |  |  |  |  |  |
| Linear-by-Linear Association | 1.813 | | 1 | | .178 |  |  |  |  |  |
| N of Valid Cases | 1685 | |  | |  |  |  |  |  |  |
| a. 0 cells (0.0%) have expected count less than 5. The minimum expected count is 7.14. | | | | | |  |  |  |  |  |

### Frequent Depression by Rural – Urban Location of Practice

| Crosstab | | | | | | |
| --- | --- | --- | --- | --- | --- | --- |
|  | | | Depressed frequently_recoded | | | Total |
|  |  |  | Neutral | Agree | Disagree |  |
| Rural_Urban_recoded | Urban_SmallCity | Count | 225 | 390 | 781 | 1396 |
|  |  | % within Rural_Urban_recoded | 16.1% | 27.9% | 55.9% | 100.0% |
|  | Rural | Count | 27 | 79 | 164 | 270 |
|  |  | % within Rural_Urban_recoded | 10.0% | 29.3% | 60.7% | 100.0% |
| Total | | Count | 252 | 469 | 945 | 1666 |
|  |  | % within Rural_Urban_recoded | 15.1% | 28.2% | 56.7% | 100.0% |

| Chi-Square Tests | | | |
| --- | --- | --- | --- |
|  | Value | df | Asymptotic Significance (2-sided) |
| Pearson Chi-Square | 6.655^a^ | 2 | .036 |
| Likelihood Ratio | 7.261 | 2 | .027 |
| Linear-by-Linear Association | 4.936 | 1 | .026 |
| N of Valid Cases | 1666 |  |  |
|  | | | |

### Aware of other Attorneys with MH Issues

| \| \| **Crosstab** \| \| \| \| \| \| \| --- \| --- \| --- \| --- \| --- \| --- \| \|  \| \| \| Are you aware of other attorneys dealing with mental health issues? \| \| Total \| \| **Yes** \| No. \| \| Practice Type: Solo_Small_Large_Prosec_DPA \| Solo Practice \| Count \| **199** \| 241 \| 440 \| \| % within Practice Type: Solo_Small_Large_Prosec_DPA \| **45.2%** \| 54.8% \| 100.0% \| \| Small to Mid-Size Firm \| Count \| **283** \| 275 \| 558 \| \| % within Practice Type: Solo_Small_Large_Prosec_DPA \| **50.7%** \| 49.3% \| 100.0% \| \| Large Firm \| Count \| **99** \| 88 \| 187 \| \| % within Practice Type: Solo_Small_Large_Prosec_DPA \| **52.9%** \| 47.1% \| 100.0% \| \| Public Defender-DPA \| Count \| **31** \| 12 \| 43 \| \| % within Practice Type: Solo_Small_Large_Prosec_DPA \| **72.1%** \| 27.9% \| 100.0% \| \| Prosecution \| Count \| **34** \| 41 \| 75 \| \| % within Practice Type: Solo_Small_Large_Prosec_DPA \| **45.3%** \| 54.7% \| 100.0% \| \| All other types \| Count \| **148** \| 125 \| 273 \| \| % within Practice Type: Solo_Small_Large_Prosec_DPA \| **54.2%** \| 45.8% \| 100.0% \| \| Total \| \| Count \| **794** \| 782 \| 1576 \| \| % within Practice Type: Solo_Small_Large_Prosec_DPA \| **50.4%** \| 49.6% \| 100.0% \|   **Chi-Square Tests** \| \| \| \| \| --- \| --- \| --- \| --- \| --- \| --- \| --- \| --- \| --- \| --- \| --- \| --- \| --- \| --- \| --- \| --- \| --- \| --- \| --- \| --- \| --- \| --- \| --- \| --- \| --- \| --- \| --- \| --- \| --- \| --- \| --- \| --- \| --- \| --- \| --- \| --- \| --- \| --- \| --- \| --- \| --- \| --- \| --- \| --- \| --- \| --- \| --- \| --- \| --- \| --- \| --- \| --- \| --- \| --- \| --- \| --- \| --- \| --- \| --- \| --- \| --- \| --- \| --- \| --- \| --- \| --- \| --- \| --- \| --- \| --- \| --- \| --- \| --- \| --- \| --- \| --- \| --- \| --- \| --- \| --- \| --- \| --- \| --- \| \|  \| Value \| df \| Asymptotic Significance (2-sided) \| \| Pearson Chi-Square \| 15.667^a^ \| 5 \| .008 \| \| Likelihood Ratio \| 15.973 \| 5 \| .007 \| \| Linear-by-Linear Association \| 2.982 \| 1 \| .084 \| \| N of Valid Cases \| 1576 \|  \|  \| \| a. 0 cells (0.0%) have expected count less than 5. The minimum expected count is 21.34. \| \| \| \|   **No Differences by Rural/Urban |
| --- | --- | --- | --- | --- | --- | --- | --- | --- | --- | --- | --- | --- | --- | --- | --- | --- | --- | --- | --- | --- | --- | --- | --- | --- | --- | --- | --- | --- | --- | --- | --- | --- | --- | --- | --- | --- | --- | --- | --- | --- | --- | --- | --- | --- | --- | --- | --- | --- | --- | --- | --- | --- | --- | --- | --- | --- | --- | --- | --- | --- | --- | --- | --- | --- | --- | --- | --- | --- | --- | --- | --- | --- | --- | --- | --- | --- | --- | --- | --- | --- | --- | --- | --- | --- | --- | --- | --- | --- | --- | --- | --- | --- | --- | --- | --- | --- | --- | --- | --- | --- | --- | --- | --- | --- | --- | --- | --- |

## Substance Use

### Drink More Now by Practice Type

| **Crosstab** | | | | | | | | |
| --- | --- | --- | --- | --- | --- | --- | --- | --- |
|  | | | | | Drink now than before pandemic_recoded | | | Total |
|  |  |  |  |  | Neutral | **Agree** | Disagree |  |
| Practice Type: Solo_Small_Large_Prosec_DPA | Solo Practice | | Count | | 86 | **92** | 296 | 474 |
|  |  |  | % within Practice Type: Solo_Small_Large_Prosec_DPA | | 18.1% | **19.4%** | 62.4% | 100.0% |
|  | Small to Mid-Size Firm | | Count | | 86 | **151** | 348 | 585 |
|  |  |  | % within Practice Type: Solo_Small_Large_Prosec_DPA | | 14.7% | **25.8%** | 59.5% | 100.0% |
|  | Large Firm | | Count | | 29 | **70** | 100 | 199 |
|  |  |  | % within Practice Type: Solo_Small_Large_Prosec_DPA | | 14.6% | **35.2%** | 50.3% | 100.0% |
|  | Public Defender-DPA | | Count | | 2 | **13** | 32 | 47 |
|  |  |  | % within Practice Type: Solo_Small_Large_Prosec_DPA | | 4.3% | **27.7%** | 68.1% | 100.0% |
|  | Prosecution | | Count | | 9 | **21** | 54 | 84 |
|  |  |  | % within Practice Type: Solo_Small_Large_Prosec_DPA | | 10.7% | **25.0%** | 64.3% | 100.0% |
|  | All other types | | Count | | 41 | **74** | 182 | 297 |
|  |  |  | % within Practice Type: Solo_Small_Large_Prosec_DPA | | 13.8% | **24.9%** | 61.3% | 100.0% |
| Total | | | Count | | 253 | **421** | 1012 | 1686 |
|  |  |  | % within Practice Type: Solo_Small_Large_Prosec_DPA | | 15.0% | **25.0%** | 60.0% | 100.0% |
| **Chi-Square Tests** | | | |  |  |  |  |  |
|  | Value | df | Asymptotic Significance (2-sided) |  |  |  |  |  |
| Pearson Chi-Square | 27.080^a^ | 10 | .003 |  |  |  |  |  |
| Likelihood Ratio | 28.146 | 10 | .002 |  |  |  |  |  |
| Linear-by-Linear Association | .626 | 1 | .429 |  |  |  |  |  |
| N of Valid Cases | 1686 |  |  |  |  |  |  |  |
| a. 0 cells (0.0%) have expected count less than 5. The minimum expected count is 7.05. | | | |  |  |  |  |  |

### Drink more now by Rural/Urban Location

| Crosstab | | | | | | |
| --- | --- | --- | --- | --- | --- | --- |
|  | | | Drink now than before pandemic_recoded | | | Total |
|  |  |  | Neutral | Agree | Disagree |  |
| Rural_Urban_recoded | Urban_SmallCity | Count | 214 | 365 | 819 | 1398 |
|  |  | % within Rural_Urban_recoded | 15.3% | 26.1% | 58.6% | 100.0% |
|  | Rural | Count | 32 | 51 | 186 | 269 |
|  |  | % within Rural_Urban_recoded | 11.9% | 19.0% | 69.1% | 100.0% |
| Total | | Count | 246 | 416 | 1005 | 1667 |
|  |  | % within Rural_Urban_recoded | 14.8% | 25.0% | 60.3% | 100.0% |

| Chi-Square Tests | | | |
| --- | --- | --- | --- |
|  | Value | df | Asymptotic Significance (2-sided) |
| Pearson Chi-Square | 10.574^a^ | 2 | .005 |
| Likelihood Ratio | 10.867 | 2 | .004 |
| Linear-by-Linear Association | 8.104 | 1 | .004 |
| N of Valid Cases | 1667 |  |  |
| a. 0 cells (0.0%) have expected count less than 5. The minimum expected count is 39.70. | | | |

### Aware of other Attorneys dealing with SA Issues by Practice Type

| **Crosstab** | | | | | |
| --- | --- | --- | --- | --- | --- |
|  | | | Are you aware of other attorneys dealing with alcohol or substance issues? | | Total |
|  |  |  | **Yes** | No |  |
| Practice Type: Solo_Small_Large_Prosec_DPA | Solo Practice | Count | **181** | 262 | 443 |
|  |  | % within Practice Type: Solo_Small_Large_Prosec_DPA | **40.9%** | 59.1% | 100.0% |
|  | Small to Mid-Size Firm | Count | **239** | 325 | 564 |
|  |  | % within Practice Type: Solo_Small_Large_Prosec_DPA | **42.4%** | 57.6% | 100.0% |
|  | Large Firm | Count | **65** | 122 | 187 |
|  |  | % within Practice Type: Solo_Small_Large_Prosec_DPA | **34.8%** | 65.2% | 100.0% |
|  | Public Defender-DPA | Count | **28** | 17 | 45 |
|  |  | % within Practice Type: Solo_Small_Large_Prosec_DPA | **62.2%** | 37.8% | 100.0% |
|  | Prosecution | Count | **34** | 44 | 78 |
|  |  | % within Practice Type: Solo_Small_Large_Prosec_DPA | **43.6%** | 56.4% | 100.0% |
|  | All other types | Count | **112** | 162 | 274 |
|  |  | % within Practice Type: Solo_Small_Large_Prosec_DPA | **40.9%** | 59.1% | 100.0% |
| Total | | Count | **659** | 932 | 1591 |
|  |  | % within Practice Type: Solo_Small_Large_Prosec_DPA | **41.4%** | 58.6% | 100.0% |

| **Chi-Square Tests** | | | |
| --- | --- | --- | --- |
|  | Value | df | Asymptotic Significance (2-sided) |
| Pearson Chi-Square | 11.899^a^ | 5 | .036 |
| Likelihood Ratio | 11.813 | 5 | .037 |
| Linear-by-Linear Association | .002 | 1 | .966 |
| N of Valid Cases | 1591 |  |  |
| a. 0 cells (0.0%) have expected count less than 5. The minimum expected count is 18.64. | | | |

**No differences by rural/urban
